# Waste foundry sand/MgFe-layered double hydroxides composite material for efficient removal of Congo red dye from aqueous solution

**DOI:** 10.1038/s41598-020-58866-y

**Published:** 2020-02-06

**Authors:** Dooraid N. Ahmed, Laith A. Naji, Ayad A. H. Faisal, Nadhir Al-Ansari, Mu. Naushad

**Affiliations:** 1 Department of Mathematics, College of Education for Pure Sciences, University of Kirkuk, Kirkuk, Iraq; 2 Department of Environmental Engineering, College of Engineering, University of Baghdad, Baghdad, Iraq; 3 Department of Civil, Environmental and Natural Resources Engineering, Lulea University of Technology, 97187 Lulea, Sweden; 4 Department of Chemistry, College of Science, King Saud University, Riyadh, 11451 Saudi Arabia

**Keywords:** Environmental monitoring, Pollution remediation

## Abstract

We aimed to obtain magnesium/iron (Mg/Fe)-layered double hydroxides (LDHs) nanoparticles-immobilized on waste foundry sand-a byproduct of the metal casting industry. XRD and FT-IR tests were applied to characterize the prepared sorbent. The results revealed that a new peak reflected LDHs nanoparticles. In addition, SEM-EDS mapping confirmed that the coating process was appropriate. Sorption tests for the interaction of this sorbent with an aqueous solution contaminated with Congo red dye revealed the efficacy of this material where the maximum adsorption capacity reached approximately 9127.08 mg/g. The pseudo-first-order and pseudo-second-order kinetic models helped to describe the sorption measurements, indicating that the physical and chemical forces governed the removal process.

## Introduction

The severity of water pollution has been resulted from the economic development adopted by human overall the world. The different industries such as textile, plastic, coating, and paper utilize the “dyes” in the different manufacturing stages^1–3^ These compounds, even at extremely low concentrations can cause a significant contamination and, consequently, the destroying of the ecosystem elements^4–6^. The continuous discharge of water contaminated with poisonous dyes to the environment can be formed a potential threat to the aquatic life and human health especially that the dyes are intrinsically toxic^7,8^. Therefore, economical and highly efficient techniques need to be developed for treatment of the contaminated water^9–11^. The presently available techniques include photocatalysis^12^, biodegradation^13^, chemical coagulation^14^, ion exchange^15–17^, and adsorption^18–22^. The adsorption is one of the most valuable methods in the treatment of the aqueous solutions because of its high efficiency, ease of operation, low cost of operation, and easy desorption^23–25^. The selection or fabrication of the adsorbents is the main operative point of adsorption, where these materials must be highly stable, low cost, environment-friendly, and definitely efficient^26,27^. Until date, literatures have been reported different reactive materials containing activated carbon, zeolite, clay, chitosan, montmorillonite, and vermiculite^7^. However, several disadvantages of these materials include the high cost, poor adsorption capacity, and low reuse rate, which limits their usage^28,29^. Thus, finding more efficient, longer durability and low cost new adsorbents are a difficult but a vital task.

Layered double hydroxide (LDH) is a type of layered anionic compound that is joined via a non-covalent bond—an interaction between non-framework interlayer anions and positively charged main lamellar. Its general formula is [M^+2^_1-x_M^+3^_×_(OH)_2_]_x_^+^[An^−^_x/n_·mH_2_O]_x_^−^, where, M^+3^ is a trivalent metal ion such as Fe^+3^, Cr^+3^, Sc^+3^, and Al^+3^; M^+2^ is divalent metal ions such as Mn^+2^, Mg^+2^, Ni^+2^, and Zn^+2^; An^−^ is an anion that balances the interlayer charge such as Cl^−^, OH^−^, NO_3_^−^, and CO_3_^−2^; x is the ratio of M^+3^/ (M^+3^ + M^+2^), varying from 0.17 to 0.33 in general^7,30,31^. The plenty of hydroxyl groups and the interlayer anions within the LDH can enhance the surface precipitation with metal ions^32^. However, the bulk/granular structure of LDHs can limit its adsorption capacity because it is produced dense multilayered stacking. The magnetic biochar can help to offer a matrix for loading LDH, thereby achieving extremely removal of pollutant^33^.

Several kinds of LDHs were synthesized until date and utilized as reactive materials to eliminate dyes from wastewater. For instance, Yuan *et al*.^34^ proved that the calcined graphene/MgAl LDH induced the removal of hexavalent chromium ion^34^. Yang *et al*.^35^ used a co-precipitation to synthesize composite sorbent consisted of core–shell Fe_3_O_4_-LDHs and their adsorption characteristic were evaluated^34^. Li *et al*.^36^ synthesized γ-AlO(OH)/Mg-Al-LDH/C with a 3D hierarchical structure and found it to have an excellent uptake capacity for Congo red dye and minocycline in wastewater. Hierarchical porous NiCo-LDH hollow dodecahedra were synthesized via MOF-templated reactions^37^. The prepared NiCo-LDH can remove heavy metals anions and organic dyestuff from wastewater due to the hierarchical porous hollow dodecahedral structure and the positive charge of their surface. Lei *et al*.^38^ certified that the Cr(VI) and Congo red can be removed effectively from wastewater by using prepared hierarchical calcined Ni/Mg/Al LDHs^38^.

Waste foundry sand (WFS) is a byproduct material resulted from the metal casting industries^39^. In the world, there are about 35,000 foundries with an annual production of 69 million metric tons of castings. Foundries produce various kinds of wastes, such as waste sand, slug, waste chemicals, wastewater, and particulate emissions where WFS forms a huge quantity among these wastes. The American Foundry Society estimated that 6.8 million tons of foundry sand^40^ was disposed in landfills, which is approximately 2/3rd of the total production. There are several drawbacks of this trend, including the early closure of the material life cycle with greater consumption of virgin resources, filling of existing landfills, and subsurface contamination in unmanaged landfill, the release of contaminated leachate, economic impact, referring in particular to logistic costs in WFS transportation (because a landfill area may not be close to the foundry), and environmental impacts. Furthermore, according to the EC regulations^41,42^, WFS is classified as a non-hazardous waste and, consequently, it has a considerable economic value, especially in terms of iron and steel. Accordingly, the principal aim of this study was to manufacture the WFS/MgFe-LDH composite sorbent for the removal of Congo red from aqueous solutions through the application of the following steps: (1) synthesis of WFS/MgFe-LDH by co-precipitation method; (2) characterization of the prepared adsorbent; and (3) the study of the adsorption performance of Congo red.

## Experimental Work

### Materials

The reactive material used in the batch experiments was WFS collected from the Nasr Company for Mechanical Industries/Baghdad/Iraq. Based on the particle size distribution curve, this waste had an effective size, median size, and uniformity coefficient of 180 µm, 320 µm, and 1.94, respectively. The major components of the WFS include SiO_2_, Al_2_O_3_, and Fe_2_O_3_ with the values of 94.36, 2.82, and 2.12%, respectively, with low percentages of other constituents such as Na_2_O of 0.24% and CaO of 0.05%. The porosity, cation exchange capacity, and bulk density of this material were 0.46, 10.94 meq/100 g, and 1440 kg/m^3^, respectively.

In addition, to simulate the water’s Congo red contamination, a solution of this dye was prepared with a concentration of 1000 mg/L and kept at the room temperature. The desired concentration of Congo red dye was obtained by dilution, and 0.1 M of HCl or NaOH were added to set the pH of this solution as required.

### Preparation of coated composite sorbent

LDHs were synthesized via the co-precipitation method at the room temperature. A series of 50 mL solution containing Mg(NO_3_)_2_·6H_2_O and FeCl_3_·6H_2_O with different molar ratios (Mg/Fe) (1/1, 2/1, 3/1, 4/1) under stirring conditions was mixed with 0.3 g WFS for manufacturing the coated sorbent and sorption tests were conducted by using 0.1 g/ 50 mL of prepared sorbent to specify the best molar ratio. Then, different dosages (0.1, 0.2, 0.3, 0.5, and 1 g/50 mL) of WFS were added to the aqueous solution of the best molar ratio specified previously to obtain five types of coated sand under the effect of WFS dosage. Again, 0.1 g/ 50 mL of prepared sorbents were applied in the sorption tests to specify the dosage of WFS suitable for manufacturing process. Drops of NaOH (2 M) and Na_2_CO_3_ (0.2 M) were added to this solution until the pH reached 7 after stirring for 1 h, and the resultant material was separated by filtration and then washed with deionized water. Thereafter, the separated solids were dried at 80 °C for 24 h, and the efficacy of the coating process was evaluated based on the sorption capacity of Congo red for the sorbents prepared under different conditions.

### Characterization of the prepared adsorbent

The crystalline structure of the prepared sorbents was examined via X-ray diffraction (XRD) (Siemens X-ray diffractometer, D8 Advance, Bruker, Germany) in Germany Laboratory/Department of Geology/College of Science/University of Baghdad. FT-IR analysis allows direct investigation of the sorption mechanisms by identifying the functional groups responsible for metal binding. Surficial morphology for coated sorbent was examined by scanning electron microscopy (SEM) equipped with an EDS (XFlash 5010; Bruker AXS Microanalysis, Berlin, Germany) operating at relative humidity of 55–60% and temperature of 21 °C to characterize the surface topography and localization of metal on the vacant sites. EDS generates digital (compositional) maps for the adopted sorbents and the brightness in the maps is related to the intensity of the pixels for the elements in the sample. This analysis can identify element concentrations of <0.1% microanalysis (Mira3; FEG-SEM Tescan, Czech).

### Batch experiments

Batch tests were performed to get the equilibrium data and to confirm the best conditions for the treatment process. These conditions included the initial pH, contact time and dosage. A series of 250 mL conical flasks were used, with each flask filled with 50 mL of an aqueous solution containing a certain concentration of Congo red dye. Different dosages of sorbent (0.01–1 g) were added to each flask, and the mixture was kept at 200 rpm stirring on an orbital shaker (Edmund Buhler SM25; German). Then, the aqueous solution was filtered through filter paper (JIAO JIE 102); a certain volume (approximately 10 mL) of the filtered solution was analyzed to measure the concentration of Congo red using an ultraviolet-visible (UV) spectrophotometer (Shimadzu Model: UV/VIS-1650) at the maximum absorption wavelength of 497 nm. The experiments for specifying the best contact time were performed by withdrawing samples periodically through the time period not exceeding 60 min. Additional tests were conducted to study the effect of initial pH at the range of 2–10 on the removal efficiency of the dye with a constant concentration of Congo red (500 mg/L). The removal efficiency was calculated as follows:

1 $$R=\frac{({C}_{o}-{C}_{e})}{{C}_{{\rm{o}}}}\times 100$$

The amount of this contaminant retained in the sorbent material, *q*_*e*_ (mg/g), can be determined by^43,44^:

2 $${q}_{e}=({C}_{o}-{C}_{e})\,\frac{{\rm{V}}}{m}$$

### Equilibrium isotherm and kinetic models for sorption data

Isotherm data for adsorption of Congo red dye by WFS/MgFe-LDH composite were modeled using the Langmuir and Freundlich isotherm models which are given as:

3 $${\bf{F}}{\bf{r}}{\bf{e}}{\bf{u}}{\bf{n}}{\bf{d}}{\bf{l}}{\bf{i}}{\bf{c}}{\bf{h}}\,{\bf{m}}{\bf{o}}{\bf{d}}{\bf{e}}{\bf{l}}:\,{q}_{e}={K}_{F}{C}_{e}^{\frac{1}{n}}$$

where *K*_*F*_ is the Freundlich constant $({\rm{mg}}/{\rm{g}}){({\rm{L}}/{\rm{mg}})}^{\frac{1}{n}}$ and $n$ is the sorption intensity^45^. This empirical isotherm is appropriate for multilayer sorption and heterogeneous surfaces^46^.

- **Langmuir model (1916):**

4 $${q}_{e}=\frac{{q}_{max}b{C}_{e}}{1+b{C}_{e}}$$

where, ${q}_{max}$ is the maximum sorption capacity (mg/g) and *b* is the affinity of the contaminant to the sorbent.

- **Pseudo first-order model:** This model is a popular expression that describe the sorption rate of dissolved compound from the aqueous solution and it can be written as follows^47^:
  5 $$\frac{{\rm{d}}q}{{\rm{d}}t}={k}_{1}({q}_{e}-{q}_{t})$$
  By applying the conditions of *q*_*t*_ = 0 at *t* = 0 and *q*_*t*_ = *q*_*e*_ at t = t, the integration of this expression can be lead to the result:
  6 $$\mathrm{ln}({q}_{e}-{q}_{t})=\,\mathrm{ln}\,{q}_{e}-{k}_{1}t$$
  The linear form of Eq. 6 can be written as below in Eq. 7 which is the Pseudo first-order model:
  7 $${q}_{t}={q}_{e}(1-{{\rm{e}}}^{-{k}_{1}t})$$
  where, *k*_1_ is the rate constant (1/min) of the Pseudo first-order.
- **Pseudo second-order model:** The derivation of this model is based on many assumptions; mainly, pollutant can be attached on the surface of reactive material as monolayer, the sorption energy remains the same for each reactive material, and no interaction can be occurred between the sorbed contaminants. This model illustrates as below^48^:

8 $$\frac{{\rm{d}}q}{{\rm{d}}t}={k}_{2}{({q}_{e}-{q}_{t})}^{2}$$

Where, *k*_2_ is the Pseudo second-order rate constant (g/mg min). Equation 8 can be integrated by applying the same conditions first-order model and the result will be as follows^48^:

9 $$\frac{1}{({q}_{e}-{q}_{t})}=\frac{1}{{q}_{e}}+{k}_{2}t$$

By arrangement, Eq. 9 can be rewritten as in Eq. 10 which represents the Pseudo second-order formula:

10 $${q}_{t}=\frac{{\rm{t}}}{(\frac{1}{{k}_{2}{q}_{e}^{2}}+\frac{t}{{q}_{e}})}$$

### Removal mechanisms

The kinetic models are not sufficient to recognize the mechanisms of the sorption process and this is required to apply the intra-particle diffusion model derived by Weber and Morris (1962)^49^. This model is an empirical formula related between the sorbed quantity and *t*^0.5^ rather than *t*, as shown below:

11 $${q}_{t}={k}_{{\rm{int}}}{t}^{0.5}+{\rm{C}}$$

where, *k*_int_ is the adsorption rate constant of the intra-particle diffusion model (mg/g min^0.5^), and C is the value of intercept that tells about the boundary layer thickness.

The linear plot between *q*_*t*_ versus *t*^0.5^ means the occurrence of intra-particle diffusion mechanism and when this plot intercept with the origin, this diffusion represents the rate limiting process; otherwise, there is other mechanisms beside the intra-particle diffusion. The straight line of intra-particle diffusion is comprised of two regions: the first one is sharper and represents the instantaneous sorption or external surface sorption, while the second region is varied gradually and the intra-particle diffusion process is rate limiting.

## Results and Discussion

### Preparation of adsorbent

**Figure 1:**
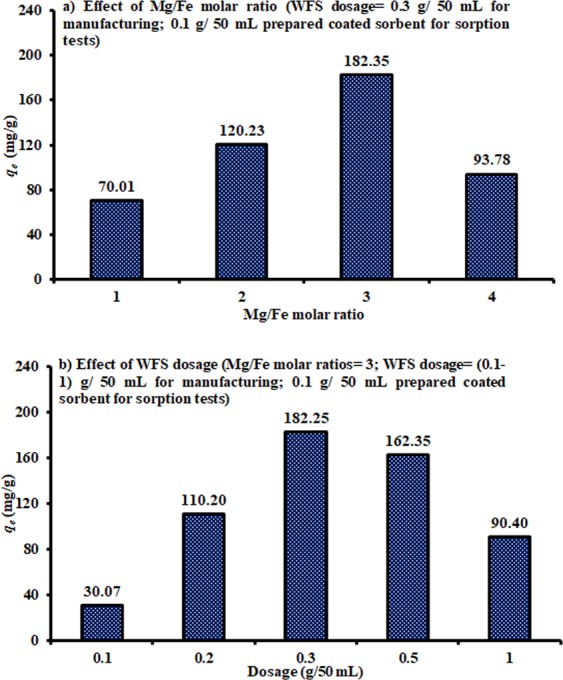
Sorption capacities of Congo red dye onto 0.1 g/50 mL coated WFS for different (**a**) Mg/Fe molar ratios and (**b**) dosages of prepared sorbent (*C*_*o*_ = 500 mg/L; pH = 3; sorption time = 3 h and agitation speed = 200 rpm).

The coated composite sorbent was developed by using certain dosage (=0.3 g/ 50 mL) of WFS mixed with different Mg/Fe molar ratios such as 1/1, 2/1, 3/1, and 4/1. The effect of the Mg/Fe molar ratios on the uptake of Congo red dye from the aqueous solutions onto 0.1 g/ 50 mL of the prepared sorbent was investigated based on the sorption capacity as an indicator for the evaluation of the effectiveness of this sorbent, as plotted in Fig. 1(a). It is clear that an increase of the Mg/Fe molar ratio causes a significant increase in the sorption capacity, and the highest value reached 182.35 mg/g occurred at a molar ratio of 3. This figure signifies that an increase in the molar ratio beyond the value of 3 can cause a remarkable decrease in the sorption capacity; accordingly, this value can be adopted to prepare an adsorbent material for the subsequent experiments.

The next step was required to specify the quantity of WFS that must be added to prepare coated composite sorbent (i.e., WFS coated with Mg/Fe-LDH), and this quantity has been changed within the range from 0.1 to 1 g/ 50 mL using Mg/Fe molar ratio of 3. By addition 0.1 g/50 mL of manufactured sorbent for sorption tests, Fig. 1(b) certified that an increase in the WFS dosage from 0.1 to 0.3 g/50 mL can increase the sorption capacity for the contaminant under consideration from 30.07 to 182.35 mg/g, respectively; then, an increase in WFS will produce a significant decrease in the sorption capacity; this behavior may be due to the limited surface of the immobilized sand. Under the same operation conditions of Fig. 1 (i.e. *C*_*o*_ = 500 mg/L, pH = 3, sorption time = 3 h and agitation speed = 200 rpm), the sorption capacity of pristine WFS with dosage of 0.1 mg/ 50 mL was measured and reached to 29 mg/g. This means that an increase in the sorption capacity of this material after coating process can be attributed to the particles of Mg/Fe-LDH precipitated on the surfaces of WFS.

### Characterization of composite sorbent

**Figure 2:**
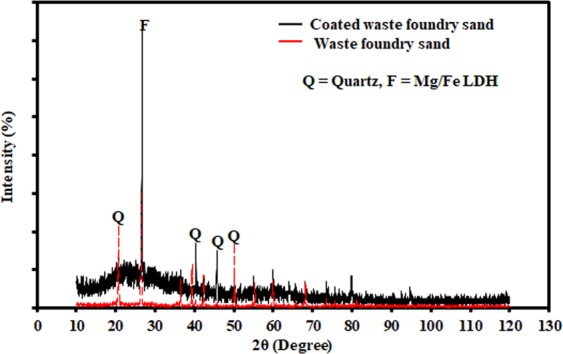
XRD data for WFS before and after coating with Mg/Fe LDH.

**Figure 3:**
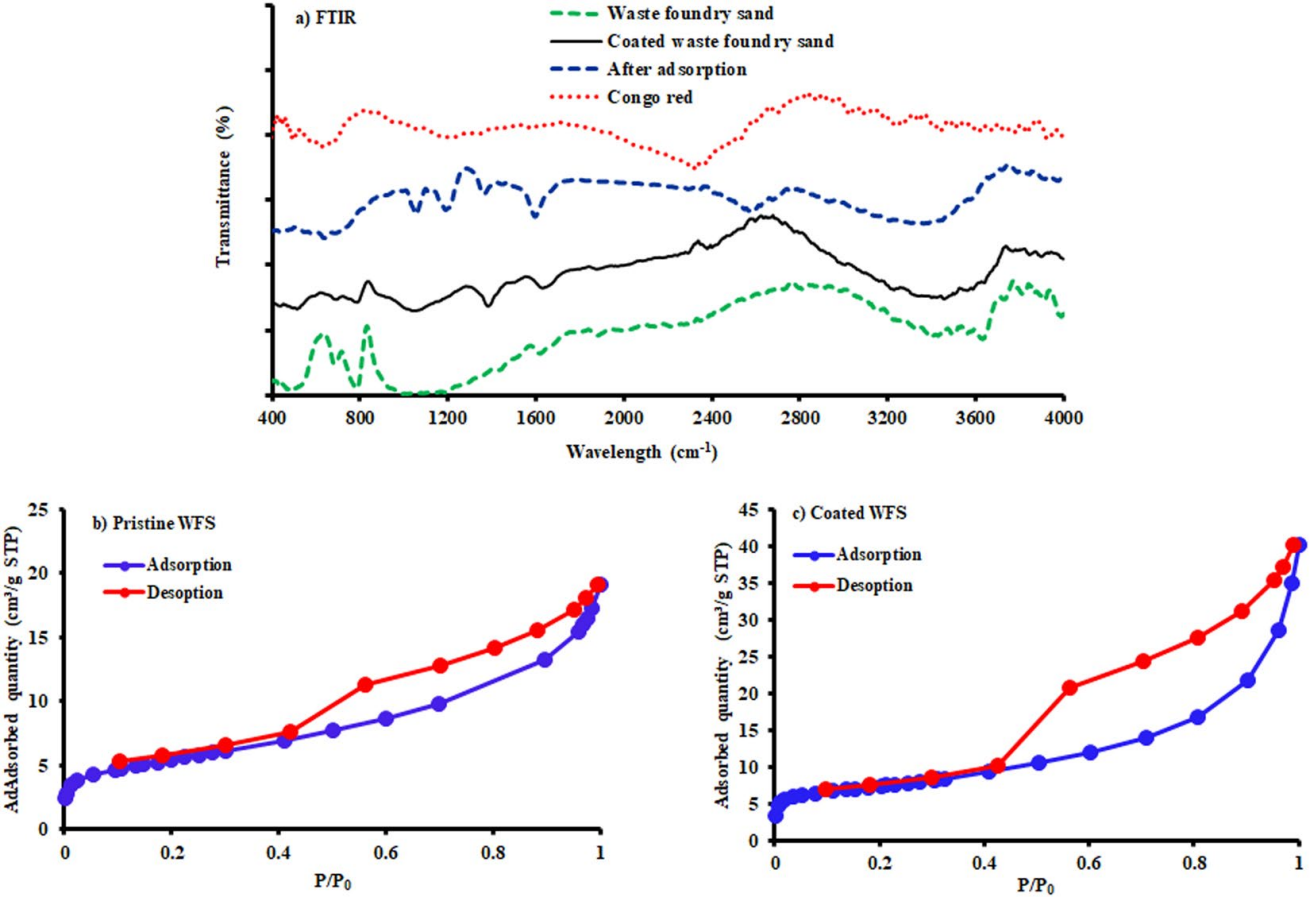
(**a**) FT-IR data for WFS, prepared composite sorbent, Congo red and composite sorbent after adsorption; (**b,c**) N_2_ adsorption-desorption isotherms.

Figure 2 depicts the XRD patterns of coated WFS, where the formation of a new peak can be seen at 26.6°, which indicates the presence of the Mg/Fe-LDH crystalline phases. This figure reveals that the synthesis of the composite sorbent consisting of WFS coated with Mg/Fe-LDH was achieved correctly. In addition, the FT-IR spectra for WFS, prepared composite sorbent, Congo red, and composite sorbent after adsorption are depicted in Fig. 3(a). The broad peak at 3390 cm^−1^ can be resulted from the stretching vibration of the -OH^23,50,51^ bonded to the inter-lamellar water, the O-H groups of the adjacent layers, and even the physically adsorbed water^38^. The peak at 1632 cm^−1^ is corresponded to the -OH groups of metal hydroxide and the bending vibration of the interlayer water (δ H-O-H)^52,53^. The peak nearest the 531 cm^−1^ is frequently accompanied to the lattice vibrations of O-M-O and M-O (where M is a metal)^36^. In comparison with WFS, new peak at 1348 cm^−1^ was observed in the composite sorbent, which can be ascribed to the asymmetric stretch absorption band and the in-of-plane vibration band of the CO_3_^−2^ ^54,55^, which suggests the successful modification of the coated sand foundry waste. After adsorption of the dye, the obtained sample displayed two evidence peaks at approximately 1080 and 1159 cm^–1^, which identical to the vibrations of the S = O and the aromatic ring of Congo red molecules, respectively^56^. The results reveal that the Red dye can be removed successfully from aqueous solutions due to the adsorption onto the surface of coated WFS.

The specific surface area and pore size have a significant effect on the adsorption ability of the prepared sorbent. Accordingly, these characteristics are measured by conducting BET test for WFS before and after the modification process. In this regard, Fig. 3(b,c) elucidates the N_2_ adsorption isotherms for samples of sorbent under consideration. For relative pressure (P/P_0_) in the range from 0.9 to 1, the adsorption-desorption isotherm curves of N_2_ for these sorbents are belonged to type IV, suggestive of mesopores type H_3_ hysteresis loop for coated WFS, which was the typical characteristic of slit-like pores formed by nanosheets^57^. At relatively low P/P. of 0.6–0.9, the type H_2_ hysteresis loop was appeared which identical to mesopores with ink-bottle shape. The outputs of these tests proved that the Langmuir surface area and micropore volume are equal to 84 m^2^/g and 0.00107 cm^3^/g for pristine WFS; however, these values are increased significantly for coated WFS to reach 135 m^2^/g and 0.00353 cm^3^/g, respectively.

**Figure 4:**
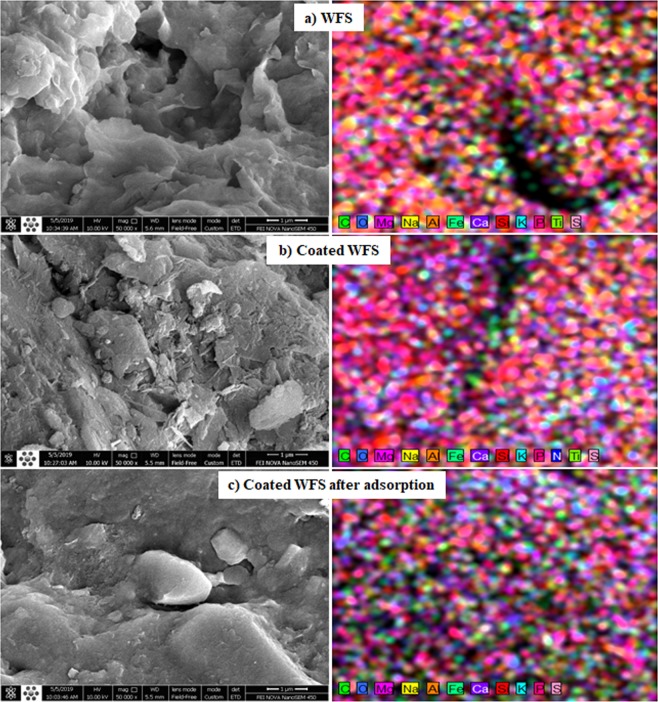
Scanning electron microscopy (SEM) images and EDS mapping for (**a**) WFS, (**b**) coated WFS and (**c**) and coated WFS after adsorption.

Figure 4 describes the surface morphology of the composite sorbent and WFS using SEM images. It is thus clear that the WFS had sharp edges and corners, rough surfaces, and compact regular pore structure. Because, the high surface area in combination with this structure, materials such as F_3_O_4_ and Mg/Fe-LDH can be planted on the surfaces of WFS. Figure 4 illustrates clearly that the magnetic iron particles are well dispersed on the WFS surface. The EDS mapping spectrum elucidates that there is a significant content of C, O, Mg, Na, Al, Fe, Ca, Si, K, P, and S in the WFS. After modification, the Fe and Mg with a purple dot increases dramatically and this revealing that the Mg/Fe-LDH was successfully planted on this waste. The S with Pink dot in the coated WFS increased significantly after the adsorption of dye, meaning that the prepared sorbent had a satisfactory uptake effect. The measurements proved that the modification of WFS by MgFe-LDH can increase its specific surface area from 19.1239 ± 0.1063 to 25.5344 ± 0.1990 cm^2^/g due to the loading of LDHs. Furthermore, the pore size decreased from 6.828 to 4.955 nm. All outputs of the characterization tests certify that the MgFe-LDH can increase the adsorption capacity of the prepared sorbent.

### Influence of operation parameters

**Figure 5:**
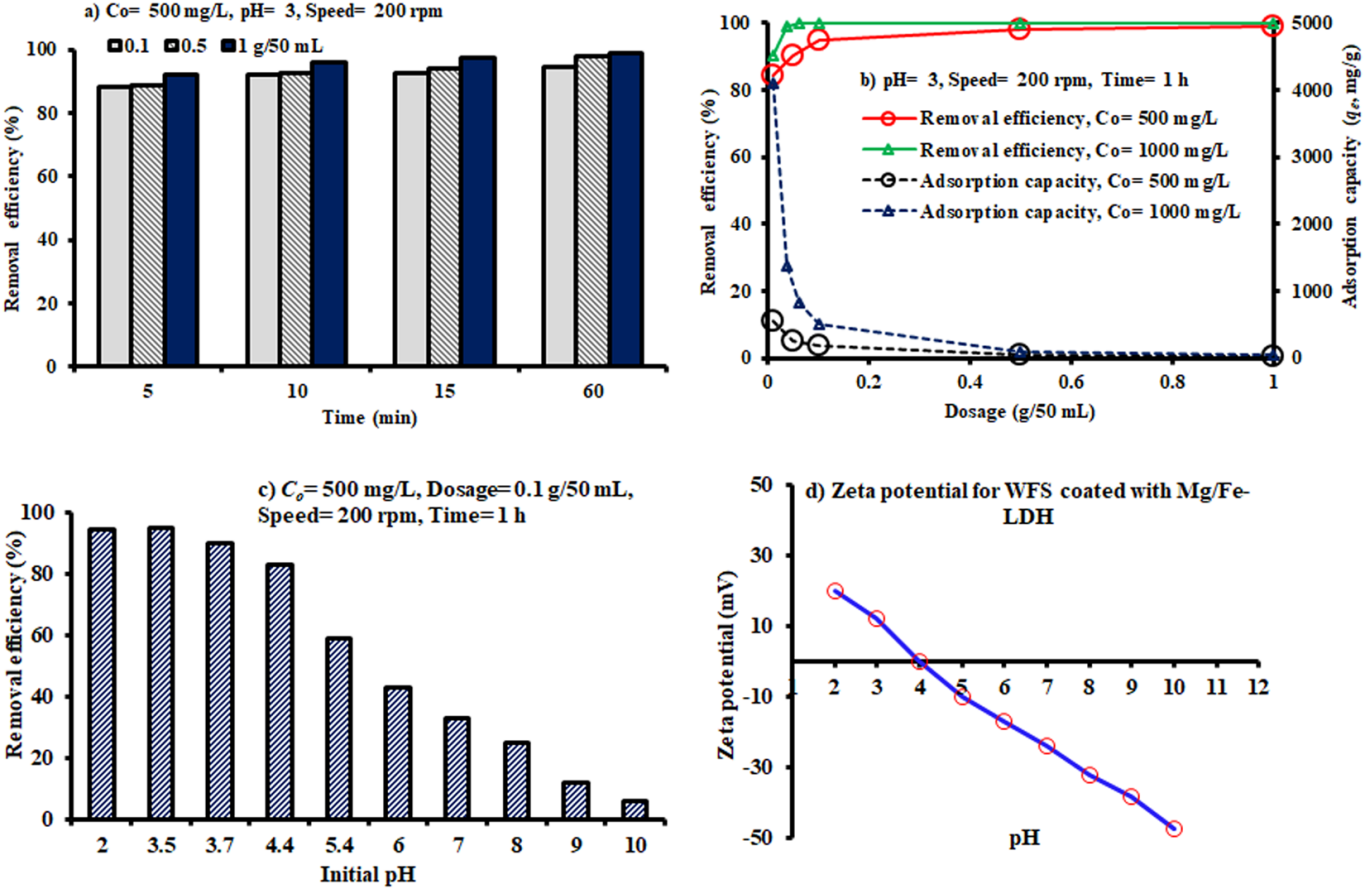
The effect of various operational parameters and zeta potential on the sorption of Congo red dye onto WFS coated with Mg/Fe-LDH.

Figure 5(a) reveals the effect of contact time on Congo red removal efficiency using different dosages (0.1, 0.5, and 1 g) of coated WFS added to 50 mL of an aqueous solution for batch tests at the room temperature. This figure demonstrates that the removal efficiency of Congo red was significantly increased with increase in the contact time. This is because the sorption process occurs in two stages: the first one is rapid sorption due to passive surface reaction like chemical (surface complexation) or physical (electrostatic) sorption onto the adsorbent, while the subsequent stage is slow owing to the occurrence of active metabolic reaction. The slower sorption was possibly due to the decrease in the sorption sites on the surface of the adsorbent. More than 97% for 1 g/50 mL was removed through 15 min and, in addition, the remaining concentrations of dye were relatively stabilized at a constant value beyond this time, which was adopted for the next sorption experiments.

Moreover, the dependence of Congo red sorption on the dosage of adsorbent was studied by changing the quantity of reactive material from 0.01 to 1 g and added to 50 mL of the contaminated solution, while keeping the other parameters as follows: *C*_*o*_ = 500 mg/L, pH = 3, shaking speed = 200 rpm, and contact time = 1 h. Figure 5(b) illustrates that the removal efficacy was improved with increasing sorbent dosage for a fixed initial concentration because higher is the dosage of the sorbent in a solution, greater is the availability of active sites^58^. With further increase in the adsorbent dosage, the adsorption capacity (*q*_*e*_) was decreased significantly, while the removal efficiency showed a steady trend.

The adsorption behavior of the prepared adsorbents for different values of pH can be affected by many parameters such as the surface charges, adsorption capacity, and the vacant active sites. Definitely there is a large number of active sites on the surface of CO_3_–LDHs contains a large number of active sites, and the removal of dye is dependable on these sites as well as the dye chemistry in the solution. The adsorption of Congo red dye onto Mg/Fe–LDH was studied at pH values 2–10 (Fig. 5c). The removal efficiency was found to be nearly constant with the highest value for pH at the range of 2–4, because the dye molecule exists as anion; then, the removal process decreased with a further increase in the pH value. A remarkable quantity of adsorption in the mentioned range means there is strong involvement of physical forces, such as hydrogen bonding, Van der Waals force, electrostatic force and hydrophopic interaction in the adsorption^59^. By adopting the same range of pH (i.e. 2–10) for sorption tests, the zeta potential was measured for composite sorbent under consideration and results are plotted in Fig. 5(d). It is obvious that the sorbent surface gets positively charged at pH < the point of zero charge (PZC) which equal to 4 and this will increase the removal of the negatively charged dye anions through electrostatic forces of attraction. At pH greater than 4, the surface of CO_3_–LDHs particles acquire a negative charge, which inhibits the adsorption of anion dye^59^. Hence, the adsorption capacity of Congo red will decrease with increasing pH. Furthermore, the diffusion rate through the pores is also affected by the zeta-potential of the samples, and the more positive zeta-potential of adsorbent is more promising for the intra-particle diffusion.

### Sorption isotherm and kinetics

**Figure 6:**
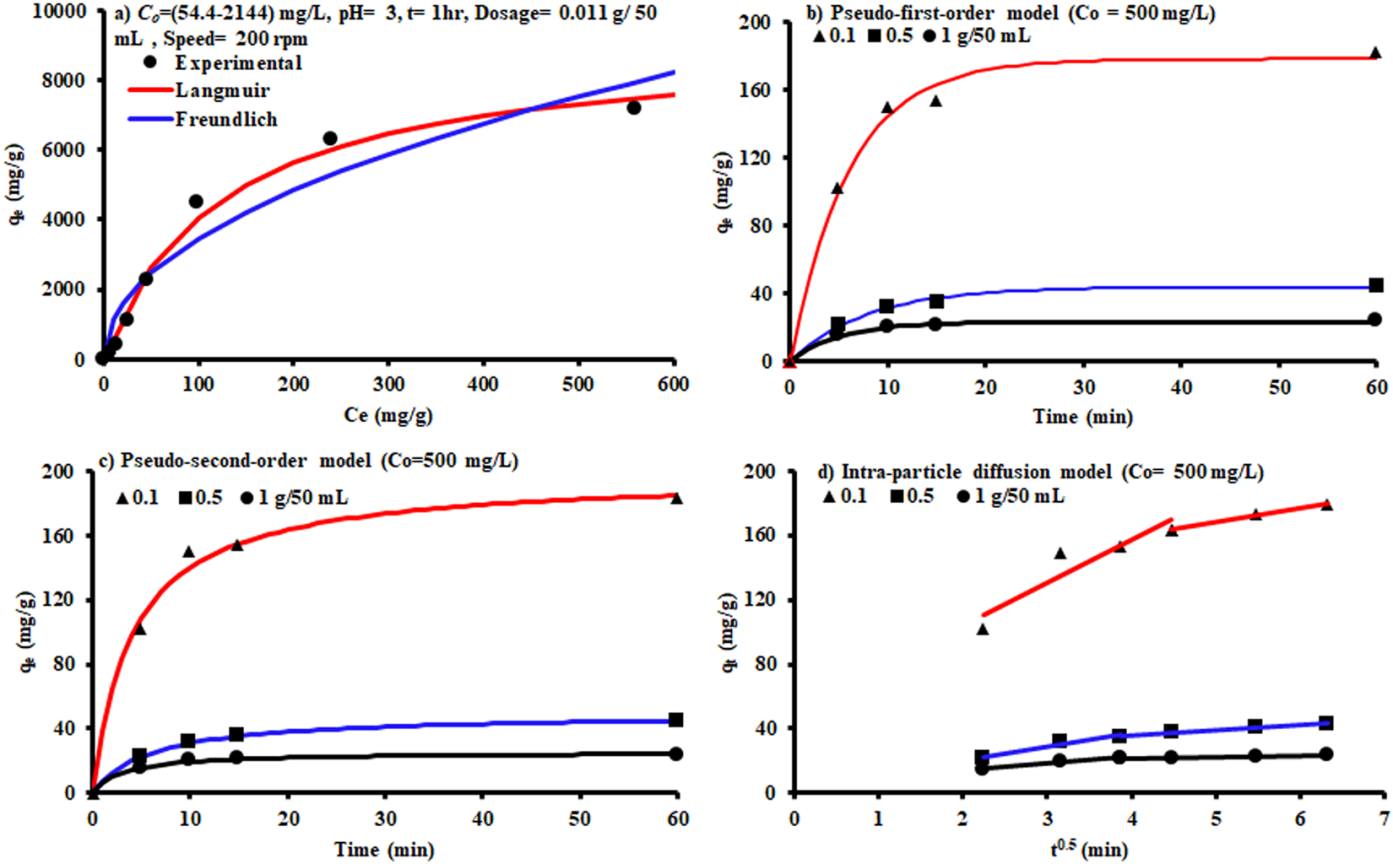
Kinetic models for sorption of Congo red dye onto WFS coated with Mg/Fe-LDH.

Freundlich and Langmuir isotherm models were applied and fitted with the sorption measurements using nonlinear fitting within the Microsoft Excel 2016. The curves of these models are shown Fig. 6(a) and their constants have been listed in Table 1. Based on coefficient of determination (R^2^) and the mentioned figure, it can be noticed that the Langmuir model is more efficient in the description of sorption data and the maximum adsorption capacity reaches to 9127.08 mg/g. Table 2 presents the maximum adsorption capacity achieved in this work (9127.08 mg/g) due to the interaction of the prepared sorbent, and the solution was contaminated with Congo red dye, which is significantly higher than that achieved by different sorbents mentioned in previous studies.

**Table 1:** Constants of isotherm models with statistical measure for sorption of Congo red dye onto WFS coated with Mg/Fe-LDH.

Isotherm model	Parameter	Value
Freundlich	*K*_*F*_ (mg/g)(L/mg)^1/*n*^	373.93
	*n*	2.07
	R^2^	0.929
Langmuir	*b* (L/mg)	0.0081
	*q*_*max*_ (mg/g)	9127.08
	R^2^	0.995

**Table 2:** Comparison of the adsorption capacity of coated WFS in comparison with other adsorbents in the previous studies for Congo red dye.

Sorbents	*q*_e_^*^(mg/g)	Reference
Coated WFS	9127.08	Present work
3D hierarchical GO-NiFe LDH	489	^60^
Flower-Like NiO microspheres	534.8	^61^
Core–shell Fe(OH)_3_@Cellulose	689.65	^62^
NiCO_2_O_4_ nanosheets	961.5	^63^
CNT/Mg(Al)O nanocomposites	1250	^35^

*Adsorption capacity.

In order to study the predominant mechanisms governing the removal of Congo red dye onto the prepared sorbent, pseudo-first-order^47^, and pseudo-second-order^48^ were fitted with the experimental kinetic data, as plotted in Fig. 6(b,c). Nonlinear regression analysis within the “Solver” option in the Microsoft Excel 2016 was used to fit the measurements with nonlinear forms of kinetic models and finding their constants. Table 3 presents the fitted constants for these models and the coefficient of determination (R^2^), which was used to evaluate the agreement between the predicted and measured values. It is apparent from the values of R^2^ and the calculated adsorption capacity in comparison with the measured ones that the both kinetic models adopted in the present study were represented the sorption of Congo red dye onto the prepared sorbent in a good manner. These findings indicated that the physical sorption and chemisorption can occur together to remove the Congo red dye from aqueous solutions.

**Table 3:** Kinetic parameters for the adsorption of Congo red dye onto WFS coated with Mg/Fe-LDH.

Kinetic model	Parameter	Dosage (g/50 mL)		
		0.1	0.5	1
Pseudo first-order	*k*_1_ (min^−1^)	0.166	0.127	0.202
	*q*_e_^*^(mg/g) *q*_eexp_^**^(mg/g)	178.15 182.35	43.85 44.67	23.27 23.75
	*R*^2^	0.959	0.979	0.983
Pseudo second-order	*k*_2_ (g/mg min)	0.001	0.003	0.013
	*q*_e_^*^(mg/g) *q*_eexp_^**^(mg/g)	197.40 182.35	49.26 44.67	25.44 23.75
	*R*^2^	0.993	0.999	0.997
Intra-particle diffusion	**First portion**			
	C	50.673	4.097	6.588
	*k*_int_ (mg/g min^0.5^)	26.603	8.300	3.987
	*R*^2^	0.860	0.963	0.940
	**Second portion**			
	C	127.04	23.97	18.088
	*k*_int_ (mg/g min^0.5^)	8.300	3.065	0.888
	*R*^2^	0.986	0.978	0.992

^*^Predicted, **Experimental.

The Weber–Morris intra-particle diffusion model was applied to define whether film diffusion or intra-particle diffusion was the rate-limiting step (Fig. 6d). The model signified that the mechanism of sorption was governed by more than one mechanism and the intra-particle diffusion was not the only rate limiting step because the plot of intra-particle diffusion not passes through the origin. This means that the external mass transfer may be predominant only in the initial stages of adsorption. The second linear portion was the gradual adsorption stage controlled by intra-particle diffusion^56^. At the initial stage, the larger slopes of the first straight line revealed that the removal rate of Congo red dye was higher because of the availability of a surface area and active sites. The lower slopes of the second portion appeared due to the reduction of the concentration gradients, indicating that the diffusion of Congo red dye in the micro-pores of sorbent takes longer, thereby reducing the rate of Congo red removal.

### Recyclability

**Figure 7:**
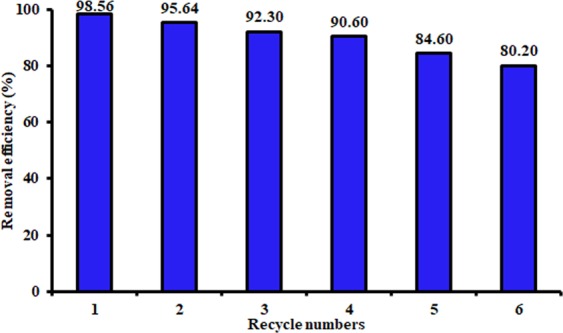
Removal efficiency of Congo red dye from the aqueous solution onto WFS coated with Mg/Fe-LDH in the recycle tests at room temperature for pH = 3, dosage = 0.1 g/50 mL and *C*_*o*_ = 500 mg/L).

Practical application of the adsorbent depends mainly on its regeneration capacity and performance stability during the adsorption process. The recyclability tests for the composite sorbent prepared in this study were implemented by applying the sorbent sample in NaOH solution with concentration of 0.1 M for 3 h to remove the sorbed Congo red molecules and to regenerate the exhausted adsorbent. Figure 7 presents the results of six-time recycle tests and it is obvious that the sorbent can be reused for removal of Congo red dye with efficiency exceeding 80%. The results indicated that the WFS coated with Mg/Fe-LDHs nanoparticle is promising sorbent in the treatment of water contaminated with anionic dye.

## Conclusions

WFS coated with Mg/Fe-LDHs nanoparticle were prepared via a precipitation procedure and its adsorption performance was evaluated by a set of batch tests for the sorption of Congo red dye from wastewater. The characterization of this sorbent was achieved by XRD, FT-IR, and SEM with EDS mapping; however, these tests certified that the surfaces of WFS were rough with sharp corners and edges, where these characteristics will enhance the coating process. The tests signified that the original WFS was modified by the planting of Mg/Fe-LDHs nanoparticles. The adsorption kinetics study revealed the effectiveness of the prepared sorbent in the elimination of Congo red dye from the aqueous medium because the maximum adsorption capacity reached a significant value (approximately 9127.08 mg/g). The pseudo first- and second-order models provided efficient description for the sorption data, which means that the physical and chemical attraction forces may be the predominant mechanisms in the removal process. In the future, WFS coated with Mg/Fe-LDHs nanoparticle opens new horizons in the use of such materials for the treatment of water contaminated with different types of contaminants by fixed bed columns and permeable reactive barrier technology.
